# National Control of Maritime Quarantine

**Published:** 1888-03

**Authors:** 


### EDITORIAL.

### NATIONAL CONTROL OF MARITIME QUARANTINE.

In a former number of the *Journal and Examiner* attention was drawn to the evident inadequacy of the quarantine arrangements in the harbor of New York. That the statement as to the danger of introduction of cholera and other infectious diseases through that important gateway, not only into New York City but to the entire country, was not an exaggeration of the facts of the case has been fully shown by the reports made to the *New York Academy of Medicine* by a committee of its members, and by a similar report by the *Board of Health* of the State of New York, as well as by the action taken by the mayor of New York. The displacement of one inefficient quarantine commissioner may remove one obstacle in the way of improvement of the service in that port, which is very desirable, as it must be considered the most dangerous, because the largest, avenue of entrance to our country for foreign immigrants and infected goods. The manifest defects in that port have served to draw attention to the existing defects in the quarantine service of our entire country, and it has been emphasized by recent importation of small-pox into San Francisco through Chinese immigration.

In October, 1887, the College of Physicians of Philadelphia appointed a committee to investigate the efficiency of our quarantine arrangements for the exclusion of cholera and other epidemic diseases. That committee has made its report, and has embodied in it a resolution to the effect that "the committee be continued, and be authorized to issue an address to the medical societies of the country," seeking their co-operation in an attempt to secure by concerted action the early adoption of a uniform and efficient quarantine at all of our exposed ports. That address, together with the report, is now being widely distributed, and may be obtained from the chairman of the committee. For the benefit of those of our readers who may not see the address, and to assist in pushing forward this very desirable movement, we give a brief summary of the main points.

From the "conclusions" reached by the committee it seems that the quarantine facilities at Baltimore and Philadelphia are utterly inadequate. Of the station at New York, the report says: "The buildings are sufficiently large and numerous, and have adequate arrangements for heating and cooking, but they are not divided into a sufficient number of small compartments to permit the strict isolation of the immigrants into small groups." The report further states that the arrangements for water-closets, drainage, washing of clothing, and for bedsteads, chairs, tables, and eating utensils were very defective.

Ample practical demonstration of the inefficiency of the arrangements at New York last fall was shown by the occurrence of cases of cholera among the quarantined immigrants, which developed much later, after the beginning of the quarantine, than the accepted period of incubation of the disease. In addition to this unequivocal evidence, the report claims, and the same statement has been made by others, that the management of the *Alesia's* passengers was worse than the arrangements.

The following summary of the "Address" mentioned is substantially in the words of the original. The weighty objections urged against maritime quarantine are:

1. The alleged failure to keep out the diseases.

2. The alleged injury to maritime trade.

The answer to the first objection is that the existing measures are grossly imperfect, and that an efficient system is theoretically and practically possible. As to the second objection, it is the ship's inhabitants, and, excluding rags, not the cargo, which carry the infection from which protection is sought. Practically, then, the present methods of independent quarantine are not efficient, and they cannot be made so. If placed in charge of a bureau of the National Government, adequate maritime quarantime measures can be established. Such a system should require:

1. That the whole matter be placed under an appropriate department of the General Government.

2. A central bureau of control established at Washington.

3. A sufficient corps of medical officers and assistants, with nurses, sanitary police, laundrymen, engineers, and officers and crews for boarding-tugs, organized at every station.

4. The erection of necessary hospital, and other buildings, wharves, disinfecting apparatus, wash-houses, latrines, etc.

5. These stations must be organized and fully equipped at every port of entry of the coast, in such a way as to meet the requirements of each port in the measure of its commerce and immigration, and the special diseases to which it is most exposed.

6. The cost of the establishment and maintenance of the National quarantine should be provided for by appropriation from the National Treasury, and not from fees exacted from vessels.

The address illustrates and defends these propositions, and closes with the following suggestions addressed to the medical societies of the country: "If your co-operation be agreed upon we would further suggest that, as a body and as individuals, you assist in influencing legislation by the following means:

1. The passage of formal resolutions recognizing the necessity of National control of maritime quarantine, and urgently recommending the matter upon the consideration of your Representatives in Congress.

2. Strenuous efforts to enlist popular sentiment in support of such legislation.

3. The enlistment of the influence of the local medical and public press."

The importance of this subject is so great that it should receive the prompt attention of the profession of the entire country. The interests involved are so universal that it would seem impossible for politicians to make a party issue upon it. Hence, there is reason to hope that if the action of this committee of the College of Physicians of Philadelphia meets with the prompt, hearty, and unanimous support which the matter deserves, it ought to be possible to secure the necessary legislation during the present session of Congress, and to have the bureau in successful operation early enough in the summer of this year to give to our country greater protection from a threatened epidemic of cholera than it possessed when the infected *Alesia* brought the disease to our doors during the past autumn, and the lateness of the season, rather than scientific and efficient quarantine, prevented the scourge from following the great lines of travel, and thus spreading throughout our country again. With the warning signal of danger so clearly shown in the harbor of New York when the cold weather of winter came to rescue the country, it would be criminal negligence that would disregard that warning and leave ports of entry yet open to invasion during the coming season.

